# Tension pneumoventricle: Reversible cause for aphasia

**DOI:** 10.5339/qmj.2021.15

**Published:** 2021-04-23

**Authors:** Nissar Shaikh, Arshad Chanda, Jazib Hassan, Asia Al-Kubaisi, Umais Momin, Abdulnasser Alyafai

**Affiliations:** ^1^Department of Anesthesia, Surgical Intensive Care & Perioperative Medicine, Hamad Medical Corporation, Doha, Qatar E-mail: nissatfirdous99@gmail.com; ^2^Department of Neurosurgery, Neuroscience Institute, Hamad Medical Corporation, Doha, Qatar; ^3^Department of Radiology, Hamad Medical Corporation, Doha, Qatar

**Keywords:** tension pneumocephalus, pneumoventricles, aphasia, external ventricular drain

## Abstract

Pneumocephalus is air in the cranium commonly seen in postcraniotomy and in head injury patients. When this air causes an increase in intracranial pressure leading to neurological deterioration, it is called tension pneumocephalus. Similarly, intraventricular air causing compression on vital centers and increasing intracranial pressure is called tension pneumoventricle, and this causes expressive aphasia, which is rarely described in the literature. This study reported a case of a traumatic cerebrospinal fluid (CSF) leak leading to tension pneumoventricle and aphasia.

Case: A young male patient sustained severe head injury and had extradural hematoma (EDH) and multiple skull and skull base fractures. EDH was drained, and he recovered and was discharged with a Glasgow coma scale score of 15. He presented to neurosurgical outpatient with CSF leak, aphasia, and loss of bowel and bladder control for a duration of three days. Computed tomography brain scan showed tension pneumoventricles, and he was started on conservative management. His general condition deteriorated, and the next day, his pupils became unequal, and Glasgow coma scale (GCS) dropped to 8/15. He was immediately taken to theater, and the air was aspirated from the ventricles, and an external ventricular drain was inserted. The patient woke up in the immediate postoperative period and started talking normally by day four.

Conclusion: Tension pneumoventricles should be considered a cause of aphasia. Immediate intervention and reduction of intracranial pressure are crucial to reverse neurological abnormality and improve patient's outcome.

## Introduction

Pneumocephalus is the presence of air in the cranial cavity, and it is a benign and common finding following craniotomy and in traumatic brain injury patients. When the amount of air in the cranial cavity increases significantly leading to neurological deterioration and functional loss or impairment due to increase in intracranial pressure (ICP), this condition is called tension pneumocephalus, and it is an emergency and life-threatening situation.^1^ The increased amount of air in the ventricles (pneumoventricle) can also cause significant pressure on the surrounding brain center and an increase in ICP and decrease in neurological status. In parallel to the term tension pneumocephalus, it is termed as tension pneumoventricles.^2^ In the literature, only few cases of reversible aphasia and motor deficit due to tension pneumocephalus following the evacuation of the chronic subdural hematoma were reported.^3,4^ This study reported a case of posttraumatic cerebrospinal fluid (CSF) leak causing tension pneumoventricle and pressure on the surrounding area leading to aphasia, brain edema, and life-threatening neurological deterioration. The patient improved with external ventricular drain (EVD) insertion and air aspiration from the ventricles.

## CASE

A young 16-year-old male patient presented to the neurosurgical outpatient department from neurorehabilitation ward with history of rhinorrhea, difficulty in talking, and inability to control his urine and bowel for two days. He suffered multiple episodes of on-and-off watery rhinorrhea since the last three days. He was involved in motor vehicular accident (passenger) two weeks back and sustained extradural hematoma (EDH) and multiple skull fractures, including the left frontoparito temporal bone, left orbit without globe injury, and skull base. He underwent evacuation of EDH and recovered, and he was shifted to rehabilitation with a Glasgow coma scale (GCS) score of 15 without any neurological abnormalities.

He was admitted to the neurosurgical ward for observation. He was awake, and pupils were equal and reactive to light. No neurological deficit was observed, but he was aphasic. Rhinorrhea was diagnosed as CSF leak and started on ceftriaxone after taking the CSF for culture and sensitivity.

His computer tomography (CT) and magnetic resonance imaging scans showed massive intraventricular pneumocephalus with ventricular distension (frontal and lateral) but failed to locate the exact site of CSF leak and demonstrated the skull base fracture. We started to nurse him in a flat position, supplemented with oxygen by face mask (6 to 8 L/minute), and shifted him to the neuro high dependency unit for close neurological monitoring.

**Figure 1.**
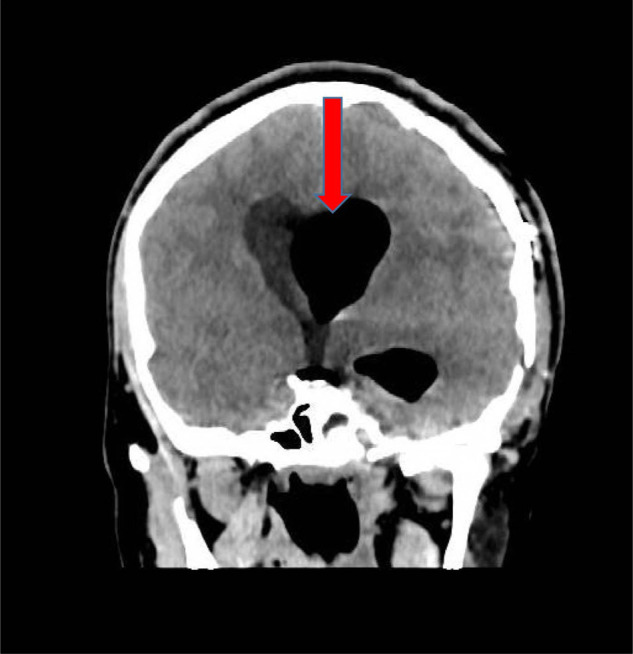
Computed tomography on admission showing tension pneumoventricles (red arrow)

On day two, he neurologically deteriorated and became confused, and his GCS decreased to 8/15. His pupils became unequal (left 5 mm and right 2 mm), and an urgent CT brain done showed increased left lateral ventricular distension due to pneumoventricle and still distended frontal ventricle with significant brain edema (Figure 1). The patient was pushed to the operating theater, and EVD was inserted. Gush of air with pressure came out during the procedure, and he was shifted to the surgical intensive care unit. In the six hours postoperative period, he was awake and still aphasic, but his pupils were equal and reactive to light. EVD continued to drain air, and he remained hemodynamically stable.

**Figure 2.**
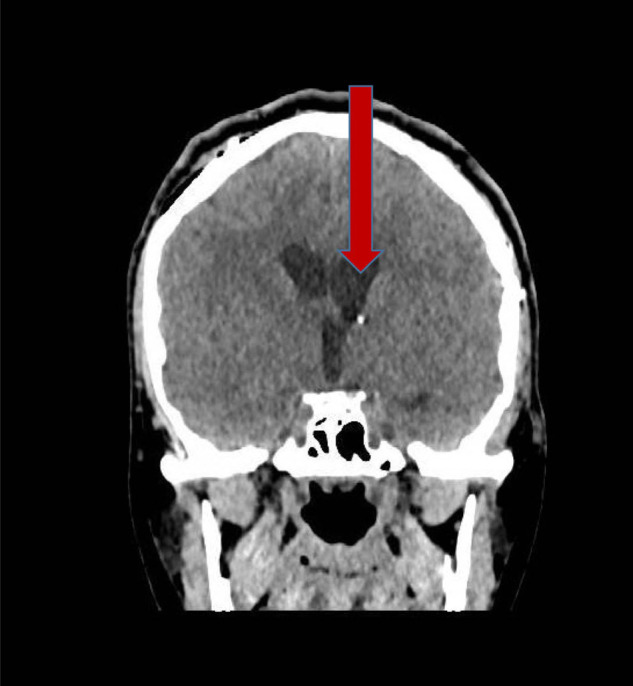
A computed tomography post-external ventricular drain (day 4) image showing resolved tension pneumoventricles (red arrow)

On day three, the patient started to murmur simple words, and supportive care was continued. By day four, he was awake and started to speak with normal articulation. The follow-up CT brain on day four showed decreased distension of the lateral ventricle with a decreased amount of pneumocephalus (Figure 2).

Pneumoventricle was completely resolved completely by day five. His speech was normal, and he regained bowel and bladder control. EVD was removed by day seven, and on day eight, he was discharged home to be followed up in neurosurgical outpatient clinics.

## Discussion

Tension pneumocephalus is a known etiology for neurological abnormality and life-threatening neurological deterioration. The reported incidence varies from 2.5% to 16%.^5^ The risk factors include chronic subdural hematoma evacuation, skull base fracture, sitting or lateral position during surgery, intraoperative diuretic use, nitrous oxide use, functioning ventricle-peritoneal shunt, and drain malpositioning.^6^ Traumatic and nontraumatic CSF leak is a risk for tension pneumocephalus.^7^ For this patient, although the exact site of CSF leak could not be located, the multiple fractures in skull base might have caused significant loss of CSF, resulting in air to enter the ventricles. All signs and symptoms manifested by this patient were due to the tension and increased pressure of air in the frontoparietal lateral ventricles, causing aphasia and later stage unisocoria, brain edema, and decreased consciousness level; hence, it was tension pneumoventricles. Only four cases of tension pneumocephalus are reported in the literature.^2,8,9^

The differential diagnosis for transient aphasia is ischemic and hemorrhagic strokes, neoplasms, cerebral abscesses, and traumatic brain contusions.^3^ This patient recovered from brain injury, and other conditions were excluded by imaging studies. In this patient, EVD insertion and air aspiration from the ventricles immediately improved his neurological status, and in the next 24 hours, when the air amount decreased significantly, he was able to talk normally, and his expressive aphasia disappeared.

The management of tension pneumocephalus and tension pneumoventricles is operative and conservative depending upon patients' neurological status. Interventions include burr hole air aspiration, craniotomy, surgical wound exploration, and lumbar subdural infusions.^7^

Conservative management includes nursing in the supine position and supplementing high flow oxygen by face mask. Oxygen supplementation helps by increasing the rate of air reabsorption from the cranium or ventricles compared with breathing on room air.^10^

The limitations of this study are that the exact CSF leak site could not be located, and it is a single case report. Future studies should be directed toward a case series or prospective study showing a correlation between tension pneumocephalus and neurological deterioration.

## Conclusion

Concluding lines are that the tension pneumoventricles following vehicular accidents should be considered a cause for neurological deterioration and aphasia. Timely intervention and immediate release of ICP, as in this case, by aspiration and continuous drainage of air from the ventricle will reverse the changes to normal.

Authors declare that they do not have any conflict of interest.
